# A Preliminary Comparison of Motor Learning Across Different Non-invasive Brain Stimulation Paradigms Shows No Consistent Modulations

**DOI:** 10.3389/fnins.2018.00253

**Published:** 2018-04-23

**Authors:** Virginia Lopez-Alonso, Sook-Lei Liew, Miguel Fernández del Olmo, Binith Cheeran, Marco Sandrini, Mitsunari Abe, Leonardo G. Cohen

**Affiliations:** ^1^Human Cortical Physiology and Neurorehabilitation Section, National Institute of Neurological Disorders and Stroke (NINDS), National Institutes of Health, Bethesda, MD, United States; ^2^Department of Physical Activity and Sport Sciences, “Center of Higher Education Alberta Giménez (CESAG)” Comillas Pontifical University, Palma, Spain; ^3^Department of Physical Education, Faculty of Sciences of Sport and Physical Education, University of A Coruña, A Coruña, Spain; ^4^Departments of Occupational Therapy, Biokinesiology, and Neurology, Stevens Neuroimaging and Informatics Institute, University of Southern California, Los Angeles, CA, United States; ^5^Molecular and Clinical Sciences Institute, St. George's, University of London, London, United Kingdom; ^6^The London Clinic, London, United Kingdom; ^7^Department of Psychology, University of Roehampton, London, United Kingdom; ^8^Faculty of Medicine, Center for Neurological Disorders, Fukushima Medical University, Fukushima, Japan

**Keywords:** non-invasive brain stimulation, motor learning, transcranial direct current stimulation (tDCS), paired associative stimulation (PAS), theta burst stimulation (TBS), power analysis

## Abstract

Non-invasive brain stimulation (NIBS) has been widely explored as a way to safely modulate brain activity and alter human performance for nearly three decades. Research using NIBS has grown exponentially within the last decade with promising results across a variety of clinical and healthy populations. However, recent work has shown high inter-individual variability and a lack of reproducibility of previous results. Here, we conducted a small preliminary study to explore the effects of three of the most commonly used excitatory NIBS paradigms over the primary motor cortex (M1) on motor learning (Sequential Visuomotor Isometric Pinch Force Tracking Task) and secondarily relate changes in motor learning to changes in cortical excitability (MEP amplitude and SICI). We compared anodal transcranial direct current stimulation (tDCS), paired associative stimulation (PAS_25_), and intermittent theta burst stimulation (iTBS), along with a sham tDCS control condition. Stimulation was applied prior to motor learning. Participants (*n* = 28) were randomized into one of the four groups and were trained on a skilled motor task. Motor learning was measured immediately after training (online), 1 day after training (consolidation), and 1 week after training (retention). We did not find consistent differential effects on motor learning or cortical excitability across groups. Within the boundaries of our small sample sizes, we then assessed effect sizes across the NIBS groups that could help power future studies. These results, which require replication with larger samples, are consistent with previous reports of small and variable effect sizes of these interventions on motor learning.

## Introduction

There has been a rising interest in the use of non-invasive brain stimulation (NIBS) to modulate brain excitability and subsequent behavior (Hallett, 2000). NIBS uses transient magnetic or electrical currents delivered to the brain through the skull (Roth et al., 1994) and is thought to affect various aspects of neuronal firing, such as synaptic firing rates and local field potentials (Reis et al., 2008). Research using NIBS has grown exponentially in the last decade, with hundreds of studies showing the potential use of NIBS in augmenting human performance across motor (Kang et al., 2015; López-Alonso et al., 2015; Buch et al., 2017a) and cognitive (Sandrini et al., 2011; Brunoni and Vanderhasselt, 2014) domains, as well as in remediating aspects of clinical disorders (Dimyan and Cohen, 2011; Liew et al., 2014; Wessel et al., 2015). However, recent research has also shown widespread interindividual variability in response to different types of NIBS applied during rest across the same individuals (Krause and Cohen Kadosh, 2014; López-Alonso et al., 2014; Buch et al., 2017b). In addition, the relationship between NIBS-induced plasticity and motor learning has been unclear (Vallence et al., 2013; López-Alonso et al., 2015). Growing studies are also showing inconsistent effects of NIBS on various behavioral tasks, but they used different types of NIBS (Kaminski et al., 2013; Minarik et al., 2015; Zhu et al., 2015; Horvath et al., 2016; McKinley et al., 2017). Here, we conducted a small preliminary study to explore the effects of three of the most commonly used excitatory NIBS paradigms over the primary motor cortex (M1) on motor learning (Sequential Visuomotor Isometric Pinch Force Tracking Task) and secondarily relate changes in motor learning to changes in cortical excitability: anodal transcranial direct current stimulation (tDCS), paired associative stimulation (PAS_25_) and intermittent theta-burst stimulation (iTBS) along with a control group (SHAM). The goal of this work was first to understand whether there were any clear relationships between each of the NIBS protocols and primarily motor learning, and second, in the event of negative effects, to estimate effect sizes to power future hypothesis-driven studies on the effects of NIBS on motor learning.

While the neural mechanisms of action of iTBS, PAS, and tDCS differ, each of these NIBS protocols has been previously shown to modulate motor-related cortical physiology and subsequent motor behavior (Nitsche and Paulus, 2000, 2001; Stefan et al., 2000, 2002; Nitsche et al., 2003; Classen et al., 2004; Huang et al., 2005; Teo et al., 2010). Cortical excitability is measured via motor-evoked potentials (MEPs) induced by single pulses of transcranial magnetic stimulation (TMS), a technique through which a transient magnetic field produces small electrical currents within the field, leading to depolarization of neurons underneath the TMS coil. The effects of neuronal firing induced by TMS over primary motor cortex can be measured by the amplitude or timing of the MEP recorded using electromyography over the stimulated contralateral muscle (Reis et al., 2008). Related, short-interval intracortical inhibition (SICI) is another measure of cortical physiology, which specifically measures activity of inhibitory interneurons in the motor cortex and is performed using two pulses of TMS (subthreshold followed by superthreshold) delivered within a short time interval (Kujirai et al., 1993; Ziemann et al., 1996; Hallett, 2000). Thus, while MEP amplitude provides information on the excitability of the motor neurons, SICI provides a complementary measure of inhibition within intracortical circuits. The collection of both allows for a more nuanced insight into the plasticity mechanisms by which NIBS may operate. Changes in cortical physiology, including both cortical excitability and inhibition, have been measured following iTBS, PAS, and tDCS (Nitsche and Paulus, 2000, 2001; Stefan et al., 2000, 2002; Huang et al., 2005). In addition, changes in motor behavior following each type of NIBS have been shown in both adaptation and skilled motor learning tasks, across all time points (online, offline, and long-term retention measures) of learning (Nitsche et al., 2003; Takeuchi et al., 2008; Ziemann and Siebner, 2008; Reis et al., 2009; Galea et al., 2010; Teo et al., 2010; Meehan et al., 2011; Stagg et al., 2011; Tanaka et al., 2011; Vollmann et al., 2013; Kim and Shin, 2014; Buch et al., 2017b). However, to our knowledge, no studies have examined the systematic differences between the effects of PAS, iTBS, and tDCS on motor learning along with the relationship between learning and cortical physiology across these three NIBS paradigms.

PAS is based on Hebb's principle that long-term potentiation is induced by spatially- and temporally-linked firing between two or more neurons (Hebb, 1949). PAS provides a precisely timed somatosensory afferent input with a TMS-induced motor efferent output to induce plasticity within that sensorimotor circuit (Stefan et al., 2000). A number of previous studies have shown that PAS effectively modulates cortical excitability (Stefan et al., 2000, 2002; Classen et al., 2004; Müller et al., 2007; Ridding and Ziemann, 2010; Conde et al., 2013). Furthermore, PAS has largely been used as a tool to probe LTP-like mechanisms of neural plasticity following motor learning (Rosenkranz et al., 2007; Cirillo et al., 2009; Fathi et al., 2010; Mang et al., 2014).

In contrast, iTBS utilizes only repeated, high-frequency, patterned trains of TMS pulses thought to induce long-lasting facilitatory effects on excitatory synaptic inputs to pyramidal tract neurons in the stimulated cortical tissue (Huang et al., 2005; Di Lazzaro et al., 2008). This results in long-lasting increases in cortical excitability following a short stimulation duration (Huang et al., 2005). iTBS over M1 has been shown to improve aspects of motor performance, such as increased acceleration of movements during a ballistic thumb abduction task (Teo et al., 2010), or improved kinematics of finger movements (Li Voti et al., 2011). On the other hand, inhibitory continuous theta-burst stimulation over M1 impaired motor performance of finger movement kinematics (Iezzi et al., 2010) and impaired implicit motor sequence learning (Wilkinson et al., 2010). In stroke patients, iTBS over ipsilesional M1 combined with motor training improved grip of the paretic limb (Ackerley et al., 2010) and improved simple reaction times of the paretic limb (Talelli et al., 2007).

Finally, unlike PAS and iTBS, tDCS does not utilize TMS but instead applies low intensity direct electrical currents that are thought to travel throughout brain tissue between the anode and cathode and modulate the local field potential of the stimulated tissue, facilitating or inhibiting the likelihood of neuronal firing (Nitsche and Paulus, 2000). As the low-amplitude electrical currents are not strong enough to immediately induce depolarization and neuronal firing as in TMS, the effects of tDCS are typically subtler and dependent on the task performed. Anodal tDCS has been widely studied in relation to motor learning, in part due to its easy application during motor learning tasks. Research has shown that anodal tDCS over M1 improves implicit motor learning (Nitsche et al., 2003), motor skill acquisition (Reis et al., 2009; Schambra et al., 2011) and motor retention (Galea et al., 2010). In clinical populations, anodal tDCS has been found to improve motor learning and motor performance in individuals after stroke (Sandrini and Cohen, 2013; Liew et al., 2014; Kang et al., 2015). However, as with each of the NIBS paradigms, it should be noted that the effects have been found to be highly variable (López-Alonso et al., 2014; Buch et al., 2017b).

The primary aim of this study was to generate preliminary data assessing the effects of these three NIBS paradigms compared to a control sham group on primarily motor learning and secondarily cortical excitability. Given the negative results, we used these data to examine the sample sizes needed to power future studies exploring these questions. We examined NIBS-related differences in motor learning effects across all groups (anodal tDCS, PAS_25_, iTBS) compared to effects of normal learning (sham control group). Notably, although all of the NIBS protocols have been shown to enhance cortical excitability and motor learning across multiple studies, we were not positive whether a comparison of the effects of these NIBS paradigms would yield positive results due to the small sample allowed by the IRB, and the likely variability among subjects which has more recently been shown (López-Alonso et al., 2014; Buch et al., 2017b; Jalali et al., 2017).

## Methods

### Subjects

Data from 28 young right-handed healthy volunteers (12/16 males/females; mean age = 27.21, *SD* = 6.93) were used for analysis. They were randomized into 4 groups receiving different NIBS protocols; sham (*n* = 7, 4 men, age = 29.57, *SD* = 2.88), tDCS (*n* = 7, 4 men, mean age = 25.86, *SD* = 2.19), PAS (*n* = 7, 1 man, mean age = 26.0, *SD* = 2.63) and iTBS (*n* = 7, 3 men, mean age = 27.43, *SD* = 3.07). All subjects had unremarkable physical and neurological history, no TMS, iTBS, tDCS, or PAS contradictions, and did not use any psychoactive medications. This study was carried out in accordance with the recommendations of the Combined Neuroscience Institutional Review Board at the National Institutes of Health with written informed consent from all subjects. All subjects gave written informed consent in accordance with the Declaration of Helsinki. The protocol was approved by the Combined Neuroscience Institutional Review Board at the National Institutes of Health.

### General procedures

**Figure 1:**
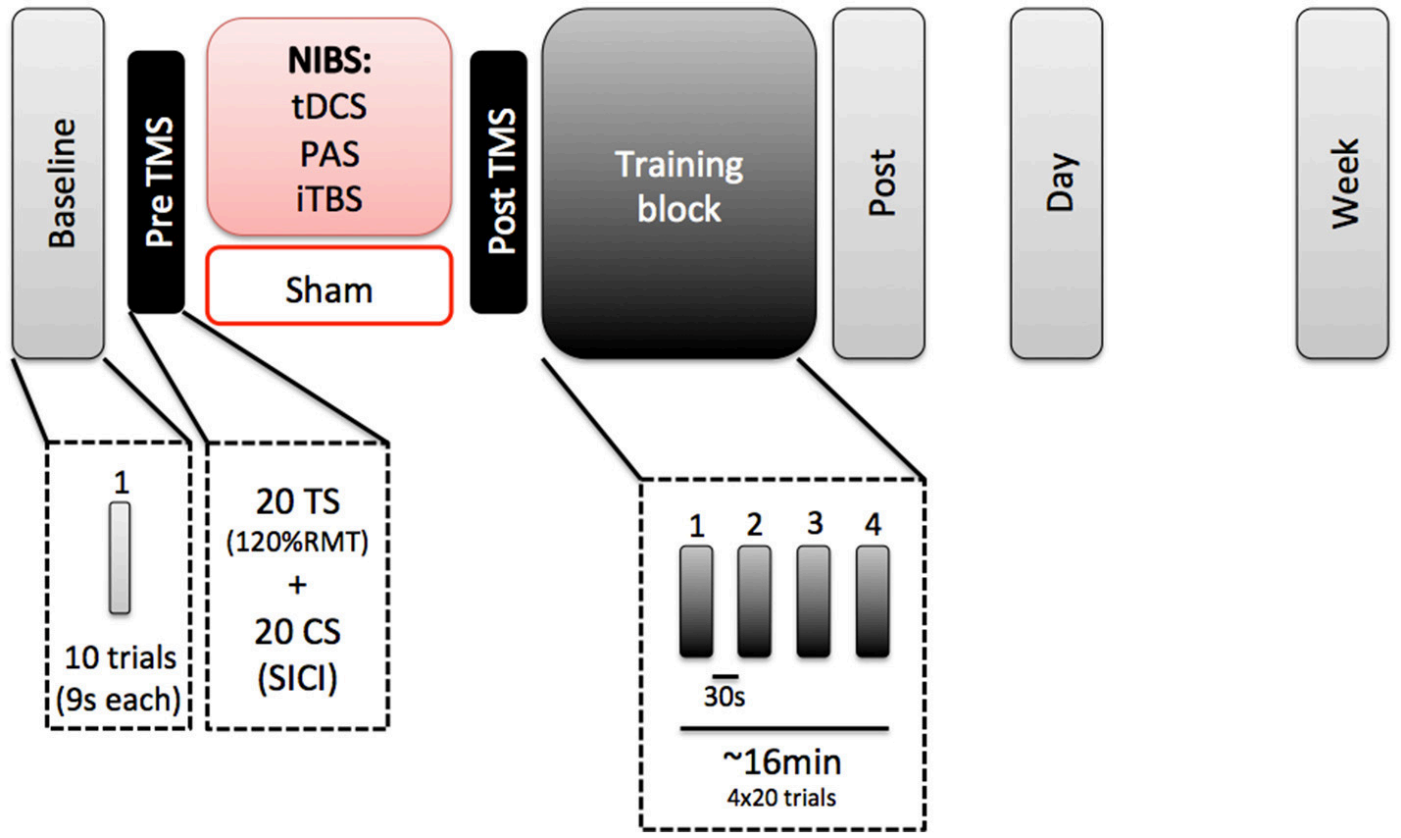
Experimental paradigm.

All subjects participated in 3 separate sessions (Figure 1). The first session took approximately 2 h and included one of the four NIBS paradigms (tDCS, TMS, PAS, or sham), followed by learning a visuomotor isometric pinch force task for approximately 16 min. We chose an offline paradigm due to the discomfort that both iTBS and PAS could induce when delivered simultaneously to motor practice. In addition, each stimulation protocol required different durations during which stimulation was delivered (e.g., iTBS was only delivered for 3 min, while PAS was delivered for roughly 30 min). Additionally, reported neuromodulatory effects for all protocols have been reported to outlast the stimulation period by 30–60 min (Stefan et al., 2000; Nitsche and Paulus, 2001; Huang et al., 2005). Sessions 2 and 3 involved only a re-test of the learning motor task. Sessions 1 and 2 were separated 24 h. Sessions 1 and 3 were separate 7 days. All sessions were tested at the same time of the day for each subject (Ridding and Ziemann, 2010).

In Session 1, subjects first performed a baseline block of the motor task (10 trials). They received the NIBS protocol, with a pre and post TMS evaluation of cortical excitability. After that, they performed a training block of the task (80 trials) and immediately after that, a post-test evaluation on the task.

#### EMG recordings

Electromyographic (EMG) traces were recorded via Ag/AgCl surface recording electrodes (7 × 4 mm recording area), placed over the right first dorsal interosseous (FDI) muscle. The active electrode was placed over the muscle belly and the reference electrode over the metacarpophalangeal joint of the index finger. Responses were acquired using a Neuropack MEB-2200 device (Nihon Kohden, Tokyo, Japan) through filters set at 10 Hz and 2 kHz with a sampling rate of 5 kHz, amplified (Micro-1401, Cambridge Electronic Devices, Cambridge, UK), and then recorded using the Signal software (Cambridge Electronic Devices, Cambridge, UK).

#### TMS procedure

TMS was delivered through a figure-of-eight coil with an outer diameter of 70 mm (Magstim Co., Whitland, Dyfeld, UK). First, we localized the hotspot for the FDI of the left M1. After that, we established the RMT. We recorded a baseline block of left M1 cortical excitability. This baseline block consisted in 20 test stimuli (TS: at an intensity of 120% of the RMT) and 20 paired pulses (a subthreshold conditioned stimulus pulse at the 80% of the RMT precedes a TS by 3 ms), randomly. With this block, we were able to measure both cortical excitability (TS MEP amplitude) and measures of cortical inhibition (SICI paired pulse). TS MEP amplitude was measured as amplitude at the peak of the MEP. The post-TMS block was identical to the pre-TMS block and used to check for changes in cortical excitability and inhibition immediately following NIBS.

#### Sequential visuomotor isometric pinch force tracking task

To measure motor learning, we used a skilled motor learning task (sequential visuomotor isometric pinch force tracking task, or SVIPT) that has previously been used to study motor skill learning over time (Abe et al., 2011). Previous work has shown that tDCS increases motor skill learning on this task (Schambra et al., 2011). SVIPT tasks have also been widely studied as a measure of motor skill learning (Cantarero et al., 2013; Wymbs et al., 2016; Mawase et al., 2017) and shown to be enhanced by tDCS over multiple days (Reis et al., 2009). Seated subjects pinched a force transducer between the right thumb pad and lateral middle phalanx of the index finger, which controlled the vertical movements of a red cursor (0.6 cm^2^). Subjects were asked to modulate their pinch force to keep the red cursor within the blue target (1.5 cm^2^). The blue target moved in a sequential pattern along a single vertical axis for 9 s during each trial. The force required to reach the target increased logarithmically with the vertical displacement. Error was defined as the vertical distance between the edges of the blue target and the red cursor at each sampled time point. Training blocks consisted of 4 blocks of 20 trials (9 s per trial) with a 30 s rest period between each block. The baseline block and test blocks (immediately after, 24 h and 7 days) consisted of 10 trials (9 s per trial).

### NIBS groups

#### PAS

PAS consisted of 90 electrical stimuli delivered at 300% of the perceptual threshold (PT) with a wave length of 200 μs over the ulnar nerve at the right wrist (cathode proximal). The electrical stimuli were paired with single TMS pulses at an interstimulus interval of 25 ms over the left hemisphere FDI hotspot at a rate of 0.05 Hz, resulting in a total protocol duration of approximately 30 min. The TMS intensity was set to evoke an MEP amplitude of 1 mV. Subjects were asked to count the number of stimuli given to ensure their attention did not vary.

#### iTBS

A Super Rapid Magstim biphasic stimulator (Magstim Co., UK) was used to deliver intermittent TBS. iTBS was applied over the left motor cortex FDI hotspot as described by Huang et al. (2005). Each burst consisted of three stimuli delivered at 80% of active motor threshold (AMT) stimulator intensity, provided at 50 Hz, with each burst repeated at 5 Hz. 2 s trains of iTBS repeated every 10 s for 20 repetitions (600 stimuli).

#### tDCS

tDCS was delivered at 1 mA for a duration of 20 min through a pair of saline-soaked sponge surface electrodes (5 × 5 cm^2^) connected to a DC stimulator (Phoresor®). The anode was placed over the hotspot of the left M1 (as determined by TMS), and the cathode was placed over the contralateral supraorbital region as in previous studies (Nitsche and Paulus, 2001; Nitsche et al., 2003, 2005). The current ramp up and ramp down were 8 s each.

#### Sham

The sham group used the same electrode montage as the tDCS group. The tDCS stimulation was turned on, with current ramped up for 8 s and then immediately ramped down for 8 s. tDCS was chosen for the sham condition, compared to sham iTBS or sham PAS, due to the ease of sham tDCS as well as previous studies showing that sham tdcs could not be reliably distinguished from active tDCS by participants.

### Statistical analyses

All statistical analyses were performed using SPSS v.20 (SPSS, Chicago, IL). To analyse changes in cortical excitability following each NIBS application, repeated measures analyses of variance (ANOVA_rm_) were conducted for the absolute values of MEP amplitude with STIMULATION (sham, tDCS, PAS, and iTBS) and TIME (baseline, post) as factors. A separate ANOVA_rm_ was conducted for absolute values of SICI amplitude with the same factors. As an exploratory analysis, differences between each NIBS group and SHAM were also compared using unpaired *t*-tests. Greenhouse-Geisser corrections were used for non-spherical data. Due to the fact that this was a pilot study, we provide both the uncorrected and corrected *p*-values when applicable. In addition, due to previous work showing inter-individual variability following NIBS (López-Alonso et al., 2014), we also calculated responders and non-responders to each NIBS protocol, reported as descriptive statistics. Responders were defined as those with an MEP change > 0 following stimulation, and non-responders as those with an MEP change < 0. While we acknowledge that this definition is quite liberal, we note that this choice is consistent with previous studies, both from our group and others (for a review, see Guerra et al., 2017).

For the motor task, the training block was analyzed in 10 trials epochs (t1, t2, …, t8). ANOVA_rm_ were conducted for the absolute and normalized values of ERROR with STIMULATION (sham, tDCS, PAS, and iTBS) and TIME (baseline, immediate post, day, and week) as factors. Normalized values of error were normalized to baseline. To better understand between-group differences at various stages of learning, one factor ANOVAs were conducted for online [(Post-Bas)/Bas], consolidation [(Day-Post)/Post], retention [(Week-Post)/Post] or training total learning [(t8-t1)/t1] between groups. Finally, to examine the relationship between motor learning and cortical excitability, correlations were run between baseline MEP (Bas) and change in MEP [(Post-Bas)/Bas] and each motor learning measure (online, consolidation, retention, and total learning). Again, as this is a pilot study, both uncorrected and corrected *p*-values are provided. Based on these results, we have also provided power analysis calculations based on this data sample for powering future studies.

Finally, we conducted a series of power analyses based on the current sample size to power future studies for selected comparisons of interest, using Cohen's d as the measure of effect size. All power analyses were conducted in G^*^Power (Faul et al., 2007) (version 3.1), using standard assumptions of α = 0.05, power (1-β) = 0.80, and two-tails for *t*-tests. Effect sizes for the primary outcome, a repeated-measures ANOVA, were calculated based on the standard formulas (Cohen, 1998):

Effect size index *f* was estimated for a repeated-measures between-within subjects ANOVA from the partial eta squared ($\eta_{p}^{2}$), which is calculated as:

$$\begin{array}{l} \eta_{p}^{2}=\;\frac{SS_{effect}}{\left(SS_{effect}+\;SS_{error}\right)} \end{array}$$

where SS_effect_ and SS_error_ are the effect and error variances. The estimation of the effect size *f* via partial eta squared was calculated as:

$$\begin{array}{l} \eta_{p}^{2}=\frac{f^{2}}{\left(1+f^{2}\right)} \end{array}$$

where *f* is the effect size, for which the equation is solved.

In addition, effect sizes for within-subjects *t*-tests (changes within a single group before and after stimulation) and independent samples *t*-tests (changes between a NIBS group and sham) were calculated as follows:

Effect size index *d*_*z*_ was calculated for a within-subjects paired samples *t*-test as follows:

$$\begin{array}{l} d_{z}=\frac{\;\mu_{z}}{\sigma_{z}} \end{array}$$

where μ_*z*_ denotes the mean difference between matched samples μ_*x*_ and μ_*y*_ and σ_*z*_ denotes the standard deviation of the difference *z*. In this calculation, the difference of the means is divided by the standard deviation of the differences.

Effect size index *d* was calculated for an independent samples *t*-test as follows:

$$\begin{array}{l} d=\;\frac{\mu_{1}-\;\mu_{2}}{\sqrt{\frac{\sigma_{1}^{2}+\sigma_{2}^{2}}{2}}} \end{array}$$

where μ_1_ and μ_2_ denote the means of each set of data, and σ_1_ and σ_2_ denote the standard deviation of each group. In this calculation, the difference of means is divided by the pooled variance between the two groups of equal sample sizes.

## Results

### Motor task performance

**Figure 2:**
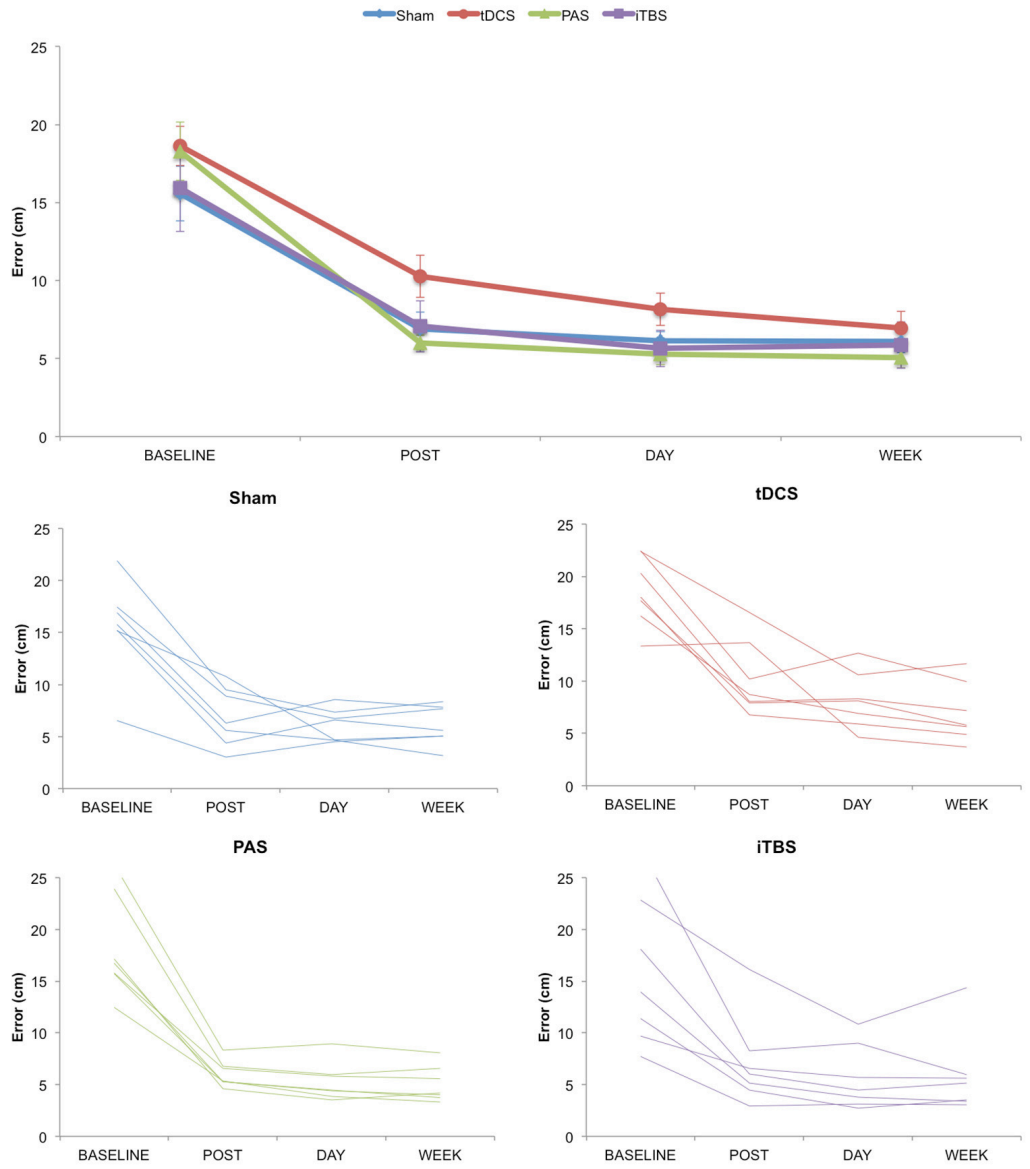
Change in motor learning as measured by error rate over time. **(Top)** Group averaged data for motor learning. **(Bottom)** Plots for Sham, tDCS, PAS25, and iTBS groups showing individual subject data. Error bars represent standard error.

We first examined changes in motor task performance across groups and time (Figure 2). The ANOVA_rm_ for absolute values of ERROR revealed a significant effect of TIME (*F* = 146.332; *p* < 0.001; η_*p*_^2^ = 0.859), with performance improved across time, but no significant effects for either GROUP (*F* = 1.086; *p* = 0.374; η_*p*_^2^ = 0.120) or TIME^*^GROUP interaction (*F* = 1.176; *p* = 0.337; η_*p*_^2^ = 0.128). Similarly, the ANOVA_rm_ for normalized values of ERROR revealed an effect of TIME (*F* = 6.149; *p* = 0.015; η_*p*_^2^ = 0.204), but no significant differences either for GROUP (*F* = 2.833; *p* = 0.060; η_*p*_^2^ = 0.261) or TIME^*^GROUP interaction (*F* = 1.082; *p* = 0.380; η_*p*_^2^ = 0.119).

We also examined changes in motor task performance across groups for each stage of motor learning. One factor ANOVAs at each stage of learning revealed no significant group differences for online (*F* = 2.331; *p* = 0.100; η_*p*_^2^ = 0.226), consolidation (*F* = 0.709; *p* = 0.556; η_*p*_^2^ = 0.081), retention (*F* = 1.503; *p* = 0.239; η_*p*_^2^ = 0.158) or training total learning (*F* = 1.100; *p* = 0.368; η_*p*_^2^ = 0.121) between groups. As an exploratory analysis, we also analyzed paired differences between NIBS groups, corrected for multiple corrections using Fisher's LSD correction. Here we only found a significant difference between tDCS and PAS on online learning (*p* = 0.014; LSD correction) and tDCS and SHAM on retention (*p* = 0.045; LSD correction).

### Cortical excitability changes

**Figure 3:**
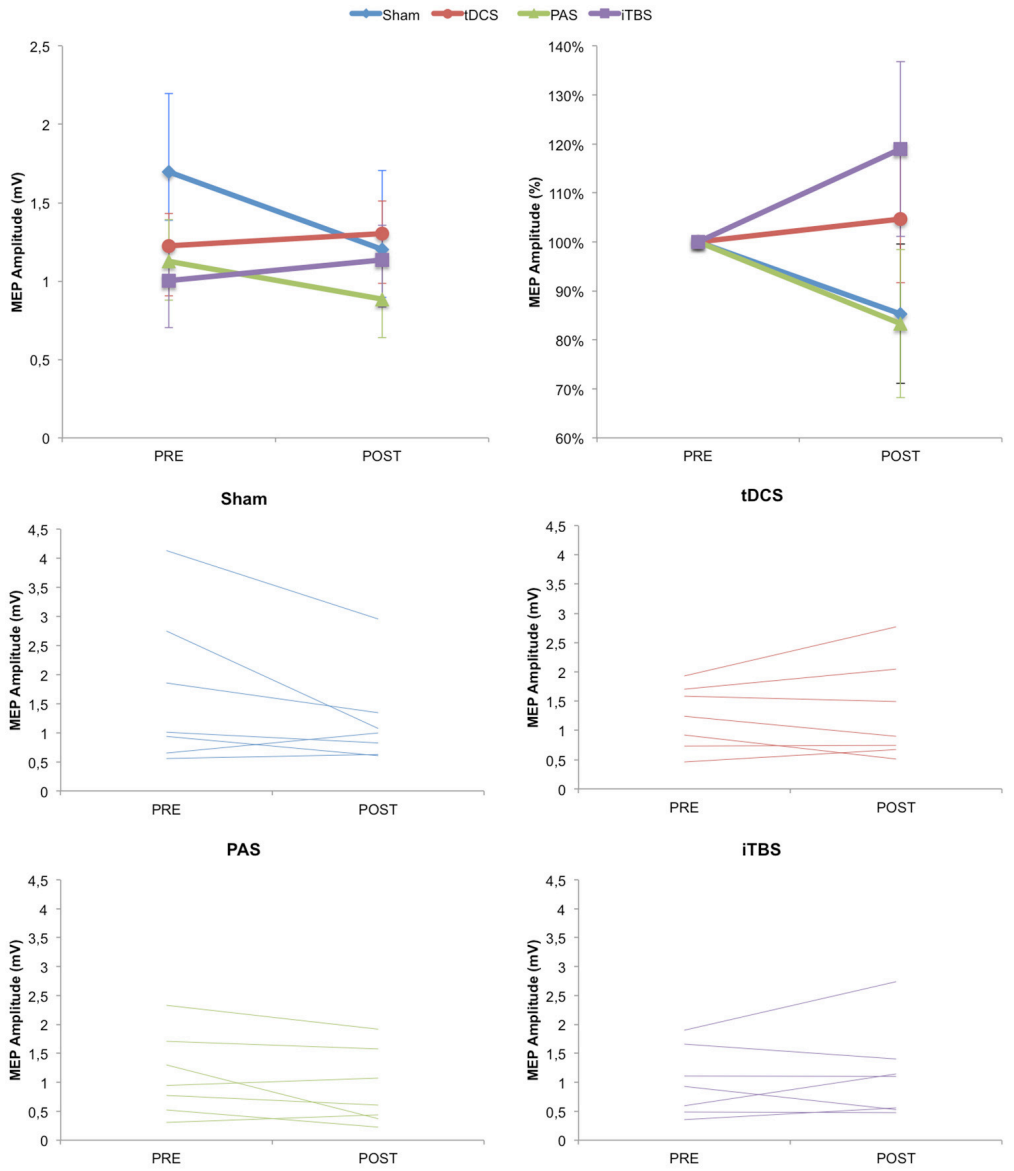
Change in cortical excitability as measured by MEP amplitude over time. **(Top)** Group averaged data for absolute MEP values and normalized MEP values. **(Bottom)** Plots for Sham, tDCS, PAS25, and iTBS groups showing individual subject data. Error bars represent standard error.

We then examined changes in cortical excitability (as measured by MEP amplitude before vs. immediately after stimulation condition) across groups. Based on previous studies, we predicted that MEP amplitude could increase following anodal tDCS, PAS_25_, and iTBS, but not after SHAM. However, the ANOVA_rm_ for MEP amplitude values revealed no effect of TIME (*F* = 1.862; *p* = 0.185; partial eta squared η_*p*_^2^ = 0.072), GROUP (*F* = 0.471; *p* = 0.705; η_*p*_^2^ = 0.056) or TIME^*^GROUP (*F* = 2.339; *p* = 0.099; η_*p*_^2^ = 0.226), although the TIME^*^GROUP interaction could be considered marginally significant (Figure 3). To examine whether there were any significantly different changes following any of the NIBS groups vs. SHAM, we also conducted unpaired *t*-tests which revealed no significant differences for any of the groups compared to SHAM (tDCS *p* = 0.661; PAS *p* = 0.295; iTBS *p* = 0.368). For reference, the significance threshold, corrected for the three comparisons, would be *p* < 0.0167.

We then examined whether there were changes in SICI as a result of NIBS across the four groups. The ANOVA_rm_ for SICI amplitude values revealed no effect of TIME (*F* = 0.152; *p* = 0.294; η_*p*_^2^ = 0.046), GROUP (*F* = 1.172; *p* = 0.341; η_*p*_^2^ = 0.128) or TIME^*^GROUP (*F* = 1.269; *p* = 0.307; η_*p*_^2^ = 0.137). Again, there were also no statistically different changes against any of the NIBS groups vs. SHAM (tDCS *p* = 0.221; PAS *p* = 0.101; iTBS *p* = 0.640).

Finally, we examined the percentage of responders vs. non-responders in each group (full data is provided in Supplementary Materials). For the anodal tDCS group, there were four responders and three non-responders (57.142% responders; mean MEP change: 0.080 ± 0.436). For the PAS_25_ group, there were two responders and five non-responders (28.571% responders; mean MEP change: −0.239 ± 0.145). For the iTBS group, there were three responders and four non-responders (42.857% responders; mean MEP change: 0.131 ± 0.182). For the SHAM group, there were 2 people whose MEPs increased following SHAM tDCS and 5 whose MEPs decreased (28.571% response; mean MEP change: −0.493 ± 0.711).

### Correlations between cortical excitability and motor learning

We finally examined whether there were any correlations between cortical excitability and motor learning. Due to the small sample, and no significant differences across groups in any of the previous measures, we collapsed this analysis across groups (*N* = 28). We analyzed the correlation of baseline MEP and change in MEP with each stage of learning (training, online, consolidation, retention). We only found a significant correlation between baseline MEP amplitude with consolidation (*R* = 0.473; *p* = 0.011) and with retention (*R* = 0.377; *p* = 0.048). However, there are no significant differences if corrected for multiple comparisons (corrected *p*-value for 8 comparisons is *p* < 0.0063). We did not find any other significant correlations between change in cortical excitability and any of the stages of learning (training learning, immediate test, consolidation, retention) across the combined groups.

### Power analyses

We also analyzed our data to estimate power for future investigations. As noted in the Methods, we used Cohen's d as the measure of effect size, with assumptions of α = 0.05, power (1-β) = 0.80, and two-tails for *t*-tests (see Table 1; for individual subject data and for additional power analyses for each NIBS group across different motor learning time points, see Supplementary Information and Supplementary Tables 1–3).

**Table 1:** Power analysis for repeated measures ANOVA with Group (tDCS, PAS, iTBS, Sham) and Time (Baseline, Immediately After, 1 Day, 1 Week).

	***F***	**Partial eta squared**	**Cohen's *f***	**Total sample size**	**Group sample size**
MEP Amplitude	2.339	0.226	0.540	48	12
Motor Learning	1.176	0.128	0.369	80	20

*A priori power analysis, ANOVA repeated measures within-between interaction, alpha = 0.05, power = 0.80, n = 7 per group*.

We first analyzed power for the repeated measured ANOVA for motor learning, with the factors of GROUP (anodal tDCS, PAS_25_, iTBS, Sham) and TIME (baseline, immediate post, day and week), which was the primary question of interest in this study. This resulted in an effect size of *f* = 0.383 (estimated from η_*p*_^2^ = 0.128) and sample size of *n* = 80, or *n* = 20 per group (Table 1). We then analyzed power for the repeated measures ANOVA for cortical excitability based on MEP amplitude, with factors of GROUP (anodal tDCS, PAS_25_, iTBS, Sham) and TIME (Baseline, Post), which was the secondary question of interest in this study. This resulted in an effect size of *f* = 0.540 (estimated from the partial eta squared η_*p*_^2^ = 0.226) and sample size of *n* = 48 total, or *n* = 12 per group (Table 1). The sample size to detect differences between groups seems relatively reasonable (12–20 subjects per group), provided one is looking at differences across these four groups.

As an exploratory measure, we then also looked at sample sizes needed for each group separately, which may be a more realistic scenario for many studies (e.g., examining pre-to-post changes in cortical excitability or motor learning following an intervention). We conducted power analyses for changes in cortical excitability as measured by MEP amplitude (Post-Stimulation > Pre-Stimulation) for each NIBS protocol (anodal tDCS, PAS_25_, iTBS; Table 2). Here we found that our preliminary data yielded the following effect sizes and subsequent sample sizes: anodal tDCS (*d* = 0.184, *n* = 234), PAS_25_ (*d* = −0.654, *n* = 21), iTBS (*d* = 0.299, *n* = 90). Notably, the sign of the effect (positive/negative) indicates the direction of the effect such that a positive effect suggests that the post-stimulation MEPs are greater than pre-stimulation MEPs, and a negative effect would show the opposite (greater pre-stimulation MEPs than post-stimulation MEPs). This would be the case for PAS_25_, in which the power is to find a higher pre-stimulation MEP than post-stimulation MEP. The average sample size needed to show pre-to-post within-subject effects following any of the NIBS protocols was *n* = 115 ± 108.68.

**Table 2:** Power analysis for each NIBS protocol (POST-PRE) on cortical excitability (MEP amplitude).

	**Mean pre**	**Mean post**	**Mean difference**	**Cohen's dz**	**Group sample size**	
Anodal tDCS	Mean	1.2252	1.3052	0.0800	0.184	234
	*SD*	0.5443	0.8442	0.4355		
PAS25	Mean	1.1243	0.8852	−0.2392	−0.654	21
	*SD*	0.7088	0.6527	0.3659		
iTBS	Mean	1.0033	1.1343	0.1310	0.299	90
	*SD*	0.5925	0.7926	0.4389		

*A priori power analysis, paired (within) samples t-test, two-tailed, alpha = 0.05, power = 0.80, n = 7 per group*.

Related, we also calculated power analyses for the common comparison of cortical excitability for one NIBS condition vs. Sham (Table 3). Here we found that our preliminary data yielded the following effect sizes and subsequent sample sizes: anodal tDCS (*d* = 0.972, *n* = 58 total with *n* = 29 per group), PAS_25_ (*d* = 0.449, *n* = 260 total with *n* = 130 per group), iTBS (*d* = 1.057, *n* = 50 total with *n* = 25 per group). The average sample size needed to show between-subject effects following any of the NIBS protocols compared to Sham was *n* = 122.67 ± 59.50.

**Table 3:** Power analysis for each NIBS protocol (POST-PRE) on cortical excitability vs. Sham.

		**Mean change NIBS**	**Mean change sham**	**Mean difference (NIBS-Sham)**	**Pooled variance**	**Cohen's d**	**Total sample size**	**Group sample size**
Anodal tDCS	Mean	0.0800	−0.4931	0.5731	0.3474	0.972	58	29
	SD	0.4355	0.7108					
PAS25	Mean	−0.2392	−0.4931	0.2539	0.3195	0.449	260	130
	SD	0.3659	0.7108					
iTBS	Mean	0.1310	−0.4931	0.6241	0.3489	1.057	50	25
	SD	0.4389	0.7108					

*A priori power analysis, independent samples t-test, two-tailed, alpha = 0.05, power = 0.80, n = 7 per group*.

Finally, we calculated power analyses for online learning, consolidation, and retention for each NIBS group compared to Sham. These results can be found in Supplementary Tables 1–3, but overall the results varied widely based on the mean differences found in the sample data.

## Discussion

In this paper, we aimed to conduct a preliminary study that would examine the effects of three commonly-used excitatory NIBS paradigms (iTBS, PAS_25_, and tDCS) on motor skill learning compared to a control sham group. We secondarily aimed to relate changes in motor learning with changes in NIBS-induced cortical excitability. We asked whether there were differences in motor skill learning and cortical excitability across the NIBS groups compared to the control group. We did not find any significant differences across any of the groups. A closer examination revealed inconsistent effects of each NIBS protocol across individuals, similar to previous findings showing high inter-individual variability across NIBS applications (López-Alonso et al., 2014). Here we focus our discussion on the potential explanations for these null effects.

Several factors could have contributed to these null results. First, we used an offline paradigm, in which each NIBS was administered prior to the motor learning task, rather than during motor training. This was chosen as each NIBS paradigm has a different stimulation timeframe (e.g., iTBS was only 3 min while PAS was 30 min) and because previously reported neuromodulatory effects outlast the stimulation period for similar periods of time across NIBS protocols (Stefan et al., 2000; Nitsche and Paulus, 2001; Huang et al., 2005). It is also conceivable that future experimental designs based on principles of metaplasticity (which considers how the history of synaptic activity influences the direction and degree of synaptic plasticity induced by a subsequent protocol) may render more effective the neuromodulation of motor learning (Abraham and Bear, 1996).

Second, as our primary focus for this investigation was changes in motor learning, rather than changes in cortical physiology, we opted to collect only one block pre/post of each MEP amplitude and SICI to avoid a long post-NIBS cortical physiology investigation that might have blurred measurement of NIBS effects on motor learning. However, it should be noted that a single block of cortical physiology measurements may be insufficient to reliably measure cortical excitability as both measures are highly variable. For future investigations with limited time for probing cortical physiology, two blocks of MEP measurements, instead of one block of MEP and one block of SICI, may improve measurement reliability (Guerra et al., 2017).

Third, as noted in the introduction, the effects of NIBS on motor behavior may be task-specific. We used a sequential visuomotor isometric pinch force task, which has previously been shown to be modulated by anodal tDCS over M1 (Reis et al., 2009; Schambra et al., 2011). In addition, we chose the SVIPT for its ability to measure motor skill learning over time without large ceiling effects. Indeed, our analyses show that all subjects were able to learn the task and improved over time. Furthermore, in our exploratory analyses, we find the anodal tDCS group performs better than the PAS group on online learning and better than the sham group on retention. However, these results are purely exploratory and would require further analysis in a larger sample. It remains to be determined if NIBS may be a more effective neuromodulator for different tasks (Classen et al., 2004; Teo et al., 2010; Li Voti et al., 2011).

Finally, perhaps the most obvious possible explanation for the negative findings we report here is our small sample size per group (*n* = 7). While we acknowledge the limited sample size, we also note that this sample size is similar to previous canonical work reporting significant increases in cortical excitability from iTBS (*n* = 9; Huang et al., 2005), tDCS (*n* = 10; Nitsche and Paulus, 2000) and PAS (subsets of *n* = 3 and *n* = 10 with significant findings; Stefan et al., 2000) as well as significant improvements in motor learning from iTBS (*n* = 10; Teo et al., 2010), tDCS (*n* = 8; Nitsche et al., 2003), and PAS (*n* = 12; Ziemann et al., 2004). Very recently, significant NIBS effects have been reported with small samples (*n* = 10 per group or less; e.g., Wade and Hammond, 2015; Watanabe et al., 2015; Casula et al., 2016; Naros et al., 2016). On the other hand, consistent with our results, several key papers have also emerged over the last few years pointing out the high inter- and intra-individual variability in NIBS effects (López-Alonso et al., 2014; Wiethoff et al., 2014; Lahr et al., 2016; Jalali et al., 2017).

Power analyses based on our results suggest that a reasonable sample of 12–20 subjects per group is needed to find significant effects in our current protocol. That is, *n* = 20 per group would be required when comparing motor learning across four NIBS groups (tDCS, PAS, iTBS, and SHAM) and four time points (baseline, post, day, week), and *n* = 12 per group would be needed to find significant MEP changes across the four NIBS groups and two time points (baseline, post). While these sample sizes do not necessarily mean that the protocol would have been successful, they do suggest that reasonable samples could be used for this type of analysis. On the other hand, power analyses based on our results for a single NIBS group (e.g., only tDCS) or a single NIBS group vs. Sham e.g., tDCS vs. Sham) were highly variable but in line with recent work which suggested large samples of *n* = 165 (PAS), *n* = 224 (tDCS), and *n* = 475 (iTBS) are needed to find significant effects for each NIBS intervention (López-Alonso et al., 2014). Taken together, these results suggest that the effects of NIBS are highly variable, and the sample size needed to detect significant changes may fluctuate largely based on each particular sample used for power analyses. This high inter-individual variability in response to NIBS and its effect on driving results is also pointed out by many recent consensus articles on the topic (Buch et al., 2017b; Guerra et al., 2017; Huang et al., 2017). In particular, it is likely that there are many other studies of similar sizes that did not find significant results and thus were not published, and that previous published studies showing significant group effects may be driven by a subset of individuals who show strong effects.

So how do we conduct studies in NIBS in light of this variability? One potential solution relies on the idea of “responder” subject recruitment. This is the idea that subjects are pre-screened with an initial NIBS session, and only those that show an initial NIBS response are included in the actual study. Research shows that intraindividual variability in response to anodal tDCS, for instance, is fair (60–69%); that is, individuals who respond to anodal tDCS once are likely to consistently show effects from tDCS in the future (López-Alonso et al., 2015). Primarily using responders for experiments has been shown to significantly reduce the sample size needed to find significant results (López-Alonso et al., 2014). This approach could be used to reduce variability in the sample and enhance the likelihood of positive findings. The critical caveats of this approach are: (1) any form of pre-screening should be clearly reported in the methods, (2) the results of studies selecting responders only precludes generalizability to a general population, and (3) the number of individuals screened vs. the number of individuals ultimately included in the study should be openly reported to allow for improved sample size calculations and transparency in the field.

In summary, our results suggest that high inter-individual variability in response to NIBS paradigms dramatically effects the sample sizes needed to show NIBS effects. These results are in line with recent proposals that emphasize the need to report more consistently both group and individual subject data, the use of pre-screening of subjects and reporting of such pre-screening steps, and a greater effort to encourage reporting of negative results, along with detailed examinations of interindividual variability.

## Author contributions

VL-A, LC, MF, and BC: Conceived and designed the study; MA: Designed the experimental stimulus; VL-A, S-LL, MS: Participated in data collection; VL-A and S-LL: Analyzed and interpreted the data and wrote the manuscript. All authors provided input on the manuscript.

### Conflict of interest statement

The authors declare that the research was conducted in the absence of any commercial or financial relationships that could be construed as a potential conflict of interest.
