# Supplementary material for: A Preliminary Comparison of Motor Learning Across Different Non-invasive Brain Stimulation Paradigms Shows No Consistent Modulations
Source: Front Neurosci. 2018 Apr 23;12:253. doi: 10.3389/fnins.2018.00253 (PMC5924807; doi:10.3389/fnins.2018.00253)
Supplement: Supplementary file 1 [file Table1.DOCX]

| **RESULTS**  In this supplementary analysis, we also calculated power analyses for online learning, consolidation, and retention for each NIBS group compared to Sham. These results can be found in Supplementary Tables 1-3 but overall the results varied widely based on the mean differences found in the sample data. The average sample size needed to show between-subject effects following any of the NIBS protocols compared to Sham across all time measurements of motor learning was n=151.75±62.64.  In these analyses, as a reduction in error meant better motor performance, negative effect sizes mean that the NIBS group showed better motor performance than the Sham group. Online learning yielded the following results: anodal tDCS (*d*=0.626, n=136 total with n=68 per group), PAS_25_ (*d*=--1.023, n=52 total with n=26 per group), iTBS (*d*=0.018, n=160,432 total with n=80,216 per group). Consolidation yielded the following results: anodal tDCS (*d*=-0.474, n=234 total with n=117 per group), PAS_25_ (*d*=-0.518, n=196 total with n=98 per group), iTBS (*d*=-0.595, n=148 total with n=74 per group). Retention yielded the following results: anodal tDCS (*d*=-0.854, n=74 total with n=37 per group), PAS_25_ (*d*=-0.534, n=186 total with n=93 per group), iTBS (*d*=-0.529, n=188 total with n=94 per group). The average sample size needed to show between-subject effects following any of the NIBS protocols compared to Sham across all time measurements of motor learning was n=151.75±62.64.  **Supplementary Table 1. Power analysis for each NIBS protocol (POST-PRE) on ONLINE LEARNING versus Sham.** | | | | | | | | |
| --- | --- | --- | --- | --- | --- | --- | --- | --- |
| *A priori* power analysis, independent samples t-test, two-tailed, alpha=0.05, power=0.80, n=7 per group. | | | | | | | | |
|  |  |  |  |  |  |  |  |  |
|  |  | **Mean Change NIBS** | **Mean Change Sham** | **Mean Difference (NIBS-Sham)** | **Pooled Variance** | **Cohen's *d*** | **Total Sample Size** | **Group Sample Size** |
| **Anodal tDCS** | Mean | -0.4319 | -0.5530 | 0.1211 | 0.0375 | **0.626** | **136** | **68** |
|  | *SD* | *0.2363* | *0.1385* |  |  |  |  |  |
|  |  |  |  |  |  |  |  |  |
| **PAS25** | Mean | -0.6627 | -0.5530 | -0.1097 | 0.0115 | **-1.023** | **52** | **26** |
|  | *SD* | *0.0620* | *0.1385* |  |  |  |  |  |
|  |  |  |  |  |  |  |  |  |
| **iTBS** | Mean | -0.5503 | -0.5530 | 0.0027 | 0.0235 | **0.018** | **160432** | **80216** |
|  | *SD* | *0.1671* | *0.1385* |  |  |  |  |  |
|  |  |  |  |  |  |  |  |  |
|  |  |  |  |  |  |  |  |  |

| **Supplementary Table 2. Power analysis for each NIBS protocol (POST-PRE) on CONSOLIDATION (1 DAY POST) versus Sham.** | | | | | | | | |
| --- | --- | --- | --- | --- | --- | --- | --- | --- |
| *A priori* power analysis, independent samples t-test, two-tailed, alpha=0.05, power=0.80, n=7 per group. | | | | | | | | |
|  |  | **Mean Change NIBS** | **Mean Change Sham** | **Mean Difference (NIBS-Sham)** | **Pooled Variance** | **Cohen's *d*** | **Total Sample Size** | **Group Sample Size** |
| **Anodal tDCS** | Mean | -0.151611244 | 0.022158916 | -0.1738 | 0.1344 | **-0.474** | **234** | **117** |
|  | *SD* | *0.295919134* | *0.425800905* |  |  |  |  |  |
|  |  |  |  |  |  |  |  |  |
| **PAS25** | Mean | -0.13918512 | 0.022158916 | -0.1613 | 0.0970 | **-0.518** | **196** | **98** |
|  | *SD* | *0.112646333* | *0.425800905* |  |  |  |  |  |
|  |  |  |  |  |  |  |  |  |
| **iTBS** | Mean | -0.173697822 | 0.022158916 | -0.1959 | 0.1085 | **-0.595** | **148** | **74** |
|  | *SD* | *0.188980872* | *0.425800905* |  |  |  |  |  |

| **Supplementary Table 3. Power analysis for each NIBS protocol (POST-PRE) on RETENTION (1 WEEK POST) versus Sham.** | | | | | | | | |
| --- | --- | --- | --- | --- | --- | --- | --- | --- |
| *A priori* power analysis, independent samples t-test, two-tailed, alpha=0.05, power=0.80, n=7 per group. | | | | | | | | |
|  |  |  |  |  |  |  |  |  |
|  |  | **Mean Change NIBS** | **Mean Change Sham** | **Mean Difference (NIBS-Sham)** | **Pooled Variance** | **Cohen's *d*** | **Total Sample Size** | **Group Sample Size** |
| **Anodal tDCS** | Mean | -0.294943858 | -0.004583237 | -0.2904 | 0.1155 | **-0.854** | **74** | **37** |
|  | *SD* | *0.224683421* | *0.424862256* |  |  |  |  |  |
|  |  |  |  |  |  |  |  |  |
| **PAS25** | Mean | -0.172858341 | -0.004583237 | -0.1683 | 0.0994 | **-0.534** | **186** | **93** |
|  | *SD* | *0.135433937* | *0.424862256* |  |  |  |  |  |
|  |  |  |  |  |  |  |  |  |
| **iTBS** | Mean | -0.170035964 | -0.004583237 | -0.1655 | 0.0980 | **-0.529** | **188** | **94** |
|  | *SD* | *0.124311978* | *0.424862256* |  |  |  |  |  |
|  |  |  |  |  |  |  |  |  |
